# Prenatal diagnosis of a 4.5-Mb deletion at chromosome 4q35.1q35.2: Case report and literature review

**DOI:** 10.1186/s13039-021-00573-y

**Published:** 2021-11-18

**Authors:** Gefei Xiao, Xianrong Qiu, Yuqiu Zhou, Gongjun Tan, Yao Shen

**Affiliations:** 1 Department of Clinical Laboratory (Institute of Medical Genetics), Zhuhai Center for Maternal and Child Healthcare, Zhuhai, China; 2 Department of Clinical Laboratory, Zhuhai Hospital of Guangdong Province Traditional Chinese Medicine, Zhuhai, China

**Keywords:** Karyotype analysis, Prenatal diagnosis, Chromosome microarray analysis, Copy number variation

## Abstract

**Objective:**

We present a genetic analysis of an asymptomatic family with a 4q terminal deletion; we also review other similar published studies and discuss the genotype–phenotype correlation.

**Methods:**

A karyotype analysis was performed on the amniotic fluid cells of a woman at 24 weeks of pregnancy and peripheral blood lymphocytes from both parents and their older son with the conventional G-banding technique. Chromosomal microarray analysis (CMA) testing was carried out for both parents and the fetus to analyze copy number variation (CNV) in the whole genome.

**Results:**

The results showed no abnormalities in the karyotypes of the father and older son, and the karyotypes of the mother and fetus were 46,XX,del(4)(q35.1) and 46,XY,del(4)(q35.1), respectively. CMA results showed a partial deletion at the 4q terminus in both the fetus and mother. The deletion region of the fetus was arr[GRCh37] 4q35.1q35.2(186,431,008_190,957,460) × 1; the loss size of the CNV was approximately 4.5 Mb and involved 14 protein-coding genes, namely, *CYP4V2, F11, FAM149A, FAT1, FRG1, FRG2, KLKB1, MTNR1A, PDLIM3, SORBS2, TLR3, TRIML1, TRIML2,* and *ZFP42*. No variation on chromosome 4 was detected in the father’s CMA results.

**Conclusion:**

Deletion of the 4q subtelomeric region is a familial variation. The arr[GRCh37] 4q35.1q35.2(186,431,008_190,957,460) region single-copy deletion did not cause obvious congenital defects or mental retardation. The application of high-resolution genetic testing technology combined with the analysis of public genetic database information can more clearly elucidate the genotype–phenotype correlation of the disease and provide support for both prenatal and postnatal genetic counseling.

## Background

Chromosomal microdeletions are caused by random genome loss during the formation of a fertilized egg and have different impacts on fetal growth and development. They can also cause mental retardation and low intelligence [1]. Flint et al. [2] found that up to 6% of unexplained mental retardation was accounted for by chromosomal subtelomeric abnormalities. Furthermore, it is generally believed that the deletion of large fragments of chromosomes leads to a poor prognosis and that the larger the lost fragment was and the more genes it contained, the more severe the clinical phenotype will be. In a large, unselected patient population study, Ravnan et al. [3] found that the unbalanced rearrangements of 10q, 4q, Yq, and X/Yp onto Xq were benign familial variants. In this report, 3 members of a family carried 4.5-Mb deletion fragments at the 4q35.1q35.2 subtelomeric region were assessed by karyotype analysis and chromosomal microarray analysis (CMA), and the possible genotypic-phenotypic correlation with the 4q terminal deletion was discussed.

## Materials and methods

### Case presentation

**Fig. 1:**
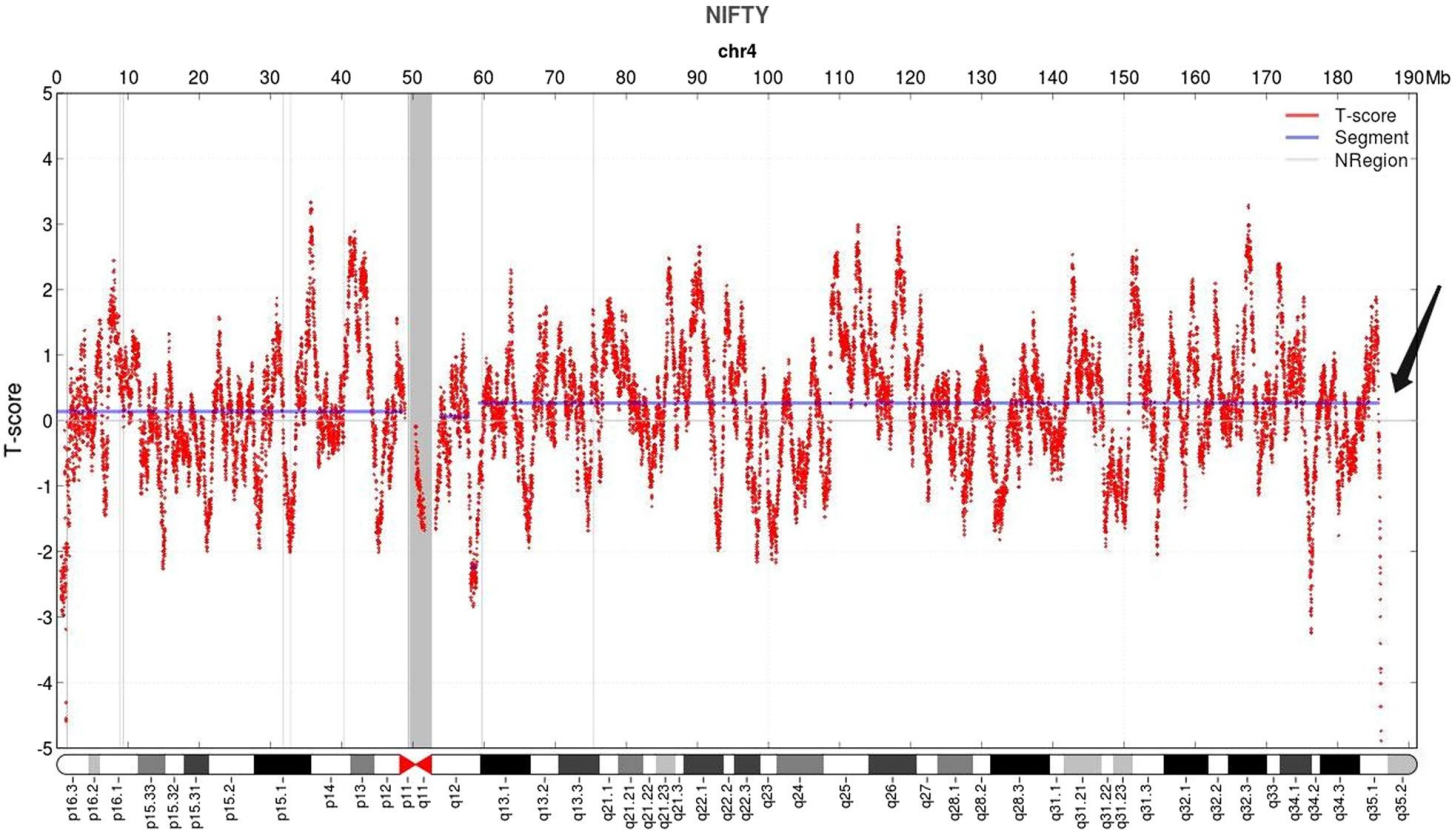
The copy number variation map of chromosome 4 by noninvasive prenatal testing. The black arrow indicates the position of the deletion at the terminus of 4q

The pregnant woman and her husband, aged 38 and 45 years, respectively, were nonconsanguineous and in good health. Family history was negative for any serious disorders. They had an 18-year-old boy who was born full-term, had a normal delivery, and was healthy. The woman had a history of a poor pregnancy one year ago. One year previously, she underwent amniocentesis to check the fetal chromosomes because of abnormal ultrasound results, which showed a possible ventricular septal defect, a focal hyperechoic region in the left ventricle, and fetal growth restriction (FGR) of approximately 2 W^+1^. The karyotype was determined to be 46,XY,del(4)(q35.1), the CMA result was arr[GRCh37] 4q35.1q35.2(186,431,008_190,957,460) × 1, and the deletion fragment size was 4.5 Mb. The couple refused parental analysis to verify whether the deletion was inherited or de novo. Finally, after receiving genetic counseling, the couple chose to terminate the pregnancy at 24 weeks. During the current pregnancy, the woman underwent noninvasive prenatal testing (NIPT) at G13^+3^ W, and the results showed a low risk for trisomy 21, 18, and 13 but a partial copy number variation (CNV) loss at the terminus of chromosome 4q as del(4q35.1-q35.2)(185,880,991_190,988,365) × 1. The deletion fragment length was approximately 5.11 Mb (Fig. 1). At G19^+1^ W of gestation, amniocentesis was performed, and amniocytes were extracted for G-banding karyotype analysis and CMA examination. At G23^+6^ W, the ultrasound results showed FGR of approximately 1w^+3^d and no other significant abnormalities. After comprehensive genetic counseling, the parents and their older son underwent cytogenetic and molecular genetic testing. This fetus was born at term without any apparent abnormal phenotype. All examinations were approved by the ethics committee, and the pregnant woman signed the informed consent form.

### Methods

#### Karyotype analysis

The pregnant woman underwent amniocentesis at G24W. Cytogenetic analysis was performed using standard, long-term culture procedures for amniocytes in two isolated flasks, each with different media. Routine chromosome analyses of peripheral blood samples from the parents and older brother were performed. Chromosomes were examined with G-banding at a resolution level of 550 bands. The karyotype was analyzed in accordance with the International System for Human Cytogenetic Nomenclature guidelines (ISCN2020).

#### Chromosomal microarray analysis

The CNVs were analyzed by CMA technology. DNA was extracted from 2 ml of peripheral blood from each parent. Fetal DNA was obtained from 10 ml of amniotic fluid. A CytoScan 750 k array (Thermo Fisher, USA) was used to detect CNVs to identify disease-causing losses and gains across the genome. Data were analyzed using Affymetrix ChAS software. The relationship between CNVs and disease was analyzed by searching public databases, including Online Mendelian Inheritance in Man®, PubMed, Decipher, the Database of Genomic Variants and the UCSC Genome Browser.

## Results

### Karyotype

**Fig. 2:**
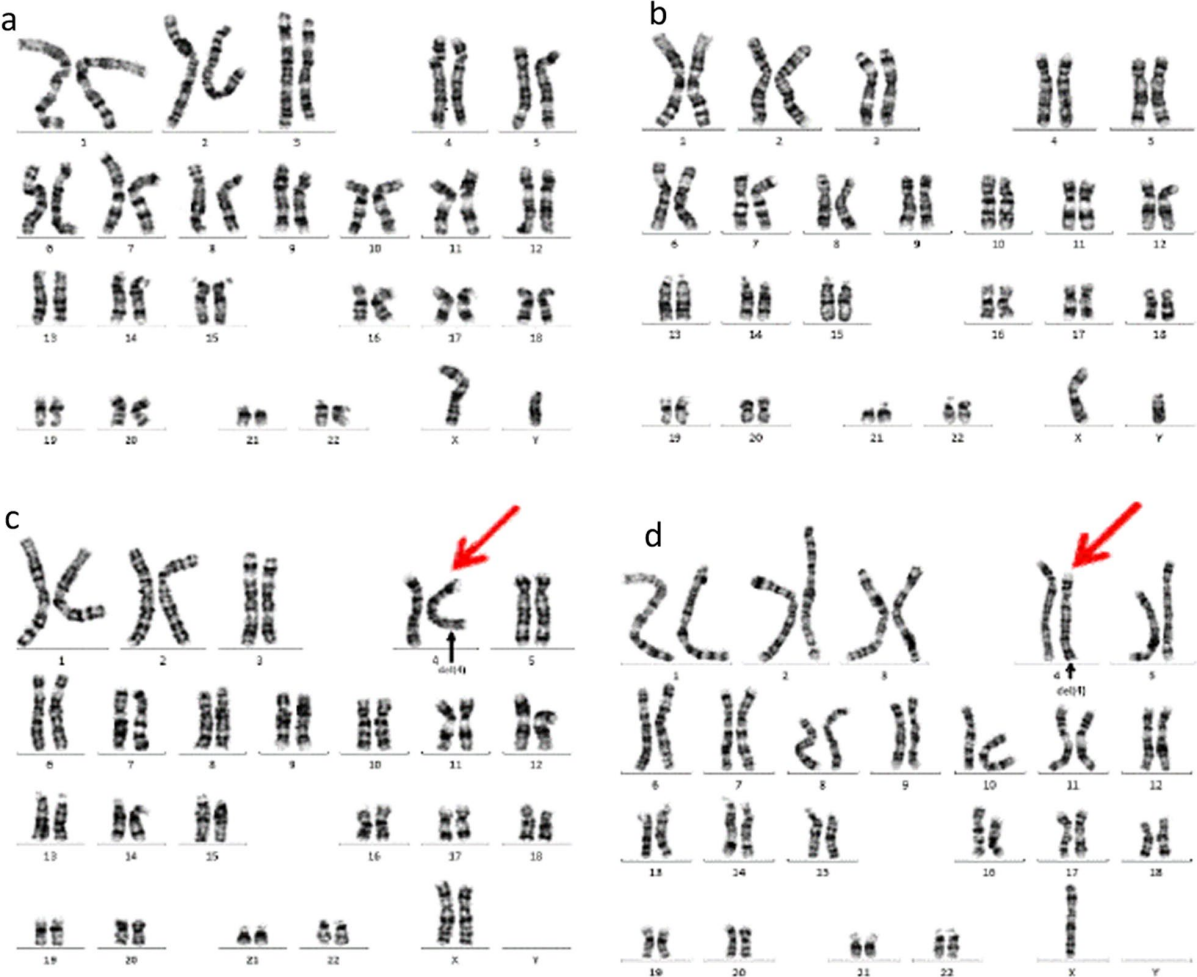
Karyotype of the family. **a** Normal karyotype of the father: 46,XY. **b** Normal karyotype of the older brother: 46,XY. **c** Abnormal karyotype of the mother: 46,XX,del(4)(q35.1). **d** Abnormal karyotype of the fetus: 46,XY,del(4)(q35.1). The red arrow indicates the abnormal chromosome 4

The karyotypes of the father and the older son were normal (Fig. 2a, b). In the analysis of the amniocytes, a segment at the terminus of 4q was found to have a copy number with a partial deletion. The same deletion was also found in the peripheral blood lymphocytes of the pregnant woman. The abnormal karyotypes were all 46,XX,del(4)(q35.1) (Fig. 2c, d), indicating that the deletion of Chr4q was inherited from the mother.

### CMA

**Fig. 3:**
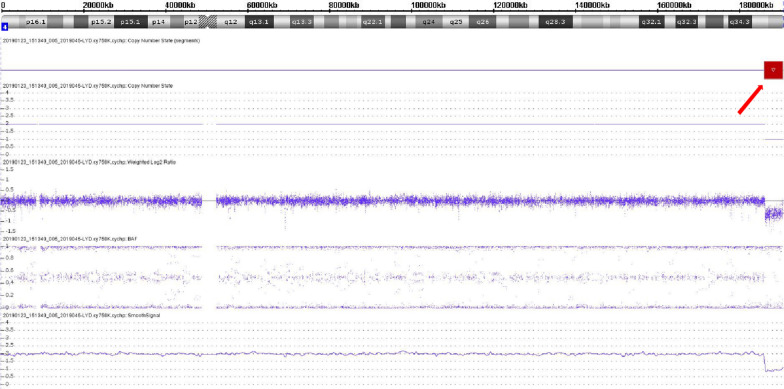
Chromosome 4 signal map from the CMA results of the fetus. The red arrow indicates the position of the deletion on the 4q terminus

CMA was performed on the fetus and both parents. Both the mother and fetus showed a 4.5-Mb loss at the 4q terminus of arr[GRCh37] 4q35.1q35.2 (186,431,008_190,957,460) × 1 (Fig. 3). This deletion region contained 14 protein-coding genes, namely, *CYP4V2, F11, FAM149A, FAT1, FRG1, FRG2, KLKB1, MTNR1A, PDLIM3, SORBS2, TLR3, TRIML1, TRIML2,* and *ZFP42* (https://www.deciphergenomics.org/search/genes?q=grch37%3A4%3A186431008-190957460). No CNV deletion was detected in the father’s chromosome 4.

## Discussion

The deletion from the 4q31 to 4q35 segment is called a distal or terminal deletion. Previous reports of the clinical phenotypes of cases with large deletions at the 4q terminus have revealed a large amount of clinical heterogeneity, with symptoms varying in severity and some individuals not having obvious phenotypes. The lengths of the deletions ranged from 1.5 Mb to 8.18 Mb, and most of them were de novo [4–8]. The most common phenotypic abnormalities in these patients are as follows: craniofacial, approximately 99%; digital, 88%; skeletal, 54%; and cardiac, 50% [9]. Disease manifestations and severity vary widely due to differences in the size of the deleted region, the exact chromosomal breaking point, and the genes located in the deleted region [10, 11].

With the advancement of high-resolution molecular diagnostic technology, such as array comparative genomic hybridization, single-nucleotide polymorphism array and next-generation sequencing technology, an increasing number of subtelomeric microdeletions have been discovered. Shao et al. [12] used CMA technology to detect 4.4% of submicroscopic chromosomal imbalances in 5380 patients, of whom 6 of 278 patients had a prior normal subtelomeric FISH analysis. Currently, more reports and databases support that 4q subtelomeric deletion does not lead to clinical symptoms (http://upd-tl.com/DB/CA/HCM/4-HM.html). Ravnan et al. [3] found 2 patients with 4q subtelomeric deletions in a data analysis of 11,688 patients referred for subtelomeric FISH testing that were inherited from their phenotypically normal fathers. The authors considered 4q subtelomeric deletion to be a family variant. Liehr [13] observed that a clinically healthy mother had a child with a developmental delay and that both had a del(4)(q34.1–34.2), while no clinical signs were observed in a person with del(4)(q35.1). Balikova et al. [14] detected a subtelomeric imbalance of 4q in a normal individual. The male propositus was born at term with normal growth parameters. However, when he was 7 years old, he was observed to have mild mental retardation but no obviously deformed appearance. Both he and his severely intellectually disabled brother carried 4q subtelomeric deletions. However, the imbalances were inherited from their mother, who had no clinical signs. The deletions were located in a region between 1.15 and 1.3 Mb. In this study, cytogenetic results showed that the pregnant woman and her two fetuses all had CNV losses at 4q35.1q35.2, but she and the second fetus did not have any clinical symptoms. Only the cardiac structure of the first fetus was abnormal, and FGR was observed on ultrasound. It may be that the presence of an unmasked recessive allele in the affected patient can also be responsible for the phenotypic difference with the parent carrier. Therefore, the CNV of the 4q subtelomeric deletion in this report that was identified in an affected proband and subsequently in an unaffected sibling and the mother are deemed probably unrelated to the clinical findings in the proband.

Another cytogenetically distinguishable 4q subtelomeric familial deletion with no obvious phenotypic effects in a mother and her two daughters was described by Yakut et al. [15]. The deletion fragment was 5.75 Mb, and the location of the region was arr[GRCh37] 4q35.1-q35.2(184,717,878_190,469,337). There were 28 protein-coding genes in the deleted region, namely, *ACSL1, ANKRD37, C4orf47, CASP3, CCDC110, CENPU, CFAP97, CYP4V2, ENPP6, F11, FAM149A, FAT1, HELT, IRF2, KLKB1, LRP2BP, MTNR1A, PDLIM3, PRIMPOL, SLC25A4, SNX25, SORBS2, STOX2, TLR3, TRIML1, TRIML2, UFSP2,* and *ZFP42* (https://www.deciphergenomics.org/search/genes?q=grch37%3A4%3A184717878-190469337). Twelve of them, *CYP4V2, F11, FAM149A, FAT1, KLKB1, MTNR1A, PDLIM3, SORBS2, TLR3, TRIML1, TRIML2*, and *ZFP42*, were the same genes involved in the deleted regions in this study. Therefore, we speculate that these genes are unlikely to be dosage-sensitive genes. More evidence is needed to support these inferences. Generally, when more than 24 protein-coding RefSeq genes are deleted, the probability that the CNV loss will cause disease increases. When there are more than 35 genes deleted, the possibility of pathogenicity is very high [16]. However, this determination can be affected by multiple factors, such as the number of genes, position of the genes, penetrance, modification of the genes, epigenetic effects, and so on. This may explain the difference in phenotypic expression between the parent and the affected child. Both our case and those of other studies advocate that more data about such cases be analyzed by high-resolution technology to clarify the deletion size and gene number thresholds that are tolerated without clinical effects.

## Conclusion

The deletion of the 4q subtelomeric region is a familial variation. The arr[GRCh37] 4q35.1q35.2(186,431,008_190,957,460) region single copy deletion does not cause obvious congenital defects or mental disorders. The use of high-resolution genetic testing technology, combined with the analysis of public genetic database information, can more clearly elucidate the genotype–phenotype correlation with disease and provide support for both prenatal and postnatal genetic counseling. This could provide more accurate diagnoses, judgment of prognosis, and clinical management for patients with different fragment sizes and region deletions at the 4q terminus.

## Data Availability

The data and materials used or analyzed during the current study are available from the corresponding author upon reasonable request.

## Abbreviations

NIPT: Noninvasive prenatal test
CNV: Copy number variation
CMA: Chromosomal microarray analysis
FGR: Fetal growth restriction
